# SE translocation gene but not zinc finger or X-linked factor is down-regulated in gastric cancer 

**Published:** 2020

**Authors:** Shiva Soleimani, Negin Nasim, Farbod Esfandi, Morteza Karimipoor, Vahid Kholghi-Oskooei, Maryam Naby Gol, Mohammad Taheri, Soudeh Ghafouri-Fard

**Affiliations:** 1 *GenIran Lab, Tashkhis Gene Pajohesh, Tehran, Iran*; 2 *Molecular Medicine Department, Biotechnology Research Center, Pasteur Institute of Iran, Tehran, Iran*; 3 *Department of Laboratory Sciences, School of Paramedical Sciences, Torbat Heydariyeh University of Medical Sciences, Torbat Heydariyeh, Iran*; 4 *Health Sciences Research Center, Torbat Heydariyeh University of Medical Sciences, Torbat Heydariyeh, Iran*; 5 *Student Research Committee, Qom University of Medical Sciences, Qom, Iran*; 6 *Urogenital Stem Cell Research Center, Shahid Beheshti University of Medical Sickness, Tehran, Iran*; 7 *Department of Medical Genetics, Shahid Beheshti University of Medical Sciences, Tehran, Iran *

**Keywords:** SE Translocation, SET, Zinc finger and X-linked factor, ZFX, Gastric cancer

## Abstract

**Aim:**

The current study aimed to identify the expression levels of *SE Translocation* (*SET*), *Zinc Finger*,* and X-Linked Factor* (*ZFX*) in gastric cancer tissues and their corresponding adjacent non-cancerous tissues (ANCTs).

**Background:**

SET has been first identified as a component of a fusion protein produced by chromosomal rearrangement in a patient with acute undifferentiated leukemia. Subsequently, multiple functions have been attributed to this gene in different disorders such as cancer and Alzheimer’s disease. The expression of SET is regulated by ZFX, a transcription factor which has a potential role in gastric cancer.

**Methods:**

In this case-control study, we evaluated the expression of *SET* and *ZFX* in gastric cancer tissues (n=28) and their corresponding ANCTs (n=28) via quantitative real-time PCR.

**Results:**

*SET1* gene was down-regulated in tumoral tissues compared with ANCTs (expression ratio=0.25, P=0.015). However, the expression of *ZFX* was similar between tumoral tissues and ANCTs (expression ratio=0.97, P=0.945). We detected a significant association between the site of primary tumor and *SET1* relative expression in tumoral tissues versus ANCTs, where this gene was down-regulated in all tumors originating from cardia. Based on the area under the receiver operating characteristic curve, the diagnostic power of transcription levels of *SET1* in gastric cancer was 0.68. Finally, we observed remarkable correlations between expression levels of *SET1* and *ZFX* both in tumoral tissues (R^2^=0.38, P<0.05) and in ANCTs (R^2^=0.23, P<0.05).

**Conclusion:**

Overall, our results imply the role of SET1 in gastric cancer and potentially functional associations between this gene and ZFX in gastric tissues.

## Introduction

 SE Translocation (SET) has been primarily recognized as a component of a fusion protein produced by chromosomal rearrangement in a patient with acute undifferentiated leukemia (1). Also known as template-activating factors I (TAF-I) beta, it has been acknowledged as a chromatin remodeling factor in human cervical cancer cells. Mainly located in the nucleus, its expression is almost constant during the cell cycle process (2). In addition, SET1 has a role in gonadal development as it can attach to certain DNA sequences to induce gene expression at all phases of xenopus oogenesis. It also modulates P450c17 expression in Leydig cells, and might induce expression of certain genes in immature oocytes (3). SET can impede the function of protein phosphatase 2A (PP2A) and change protein phosphorylation thus modifying many cellular functions (4, 5). SET is also involved in the pathogenesis of Alzheimer’s disease; Chasseigneaux et al. reported an association between the cytoplasmic localization of SET and the reduction of methylated PP2A levels causing diminished PP2A activity and tau hyperphosphorylation (6). The role of SET in the carcinogenesis has been demonstrated through cell line studies. They have reported that stable inhibition of SET has suppressed cell growth, migration, and invasion of breast cancer cells and inhibited matrix metalloproteinase 9 (MMP-9) expression (5). 

Notably, Zinc Finger and X-Linked Factor (ZFX) has been shown to regulate the expression of SET through binding with its promoter (7). This transcription factor has a potential role in the pathogenesis of gastric cancer with Nikpour et al. reporting its differential expression in gastric cancer tissues and remarkable association between its expression and tumor subtypes and histological grade. Expression of ZFX increased in diffused-type and grade III gastric cancer specimens tissues (8).

Based on the reported role of SET in carcinogenesis, associations between SET and ZFX expressions and established function of ZFX in gastric cancer, we designed the current investigation to compare the expression of these two genes in gastric cancer tissues and their paired adjacent non-cancerous tissues (ANCTs). 

## Methods

**Patients**

Expression assays were performed on 56 paired tissue specimens acquired from 28 patients with histopathologically-proven gastric cancer. ANCTs devoid of tumor cells were considered as control tissues. All tissues were obtained from Cancer Research Institute, Imam Khomeini Hospital, Tehran, Iran during April 2017-April 2018. The tissues were collected prior to chemo/radiotherapy. The study protocol was approved by the local ethics committee. All patients signed written informed consent forms. Tumor Node Metastasis (TNM) staging was based on the system developed by the American Joint Committee on Cancer (9). 

**Expression assays**

For the purpose of expression assay, the total RNA was extracted from tumoral specimens and ANCTs using the TRIzol™ Chemical (Invitrogen, Carlsbad, CA, USA) based on the protocol stated by the company. cDNA was produced from approximately 75 ng of RNA using the cDNA Reverse Transcription Kit (Applied Biosystems, Foster City, CA, USA). The relative transcription of *SET1* and *ZFX* was measured in each sample in the Rotor Gene 6000 Real-Time PCR system. Transcript levels were normalized to transcripts of *B2M*. Experiments were performed using TaqMan® Universal PCR Master Mix (Applied Biosystems, Foster City, CA). The sequences of primers are reported in Table 1.

**Statistical Analysis**

Statistical analyses were performed in SPSS v.20 (IBM Corp., Armonk, NY, USA). The difference in transcript values of genes between tumoral tissues and ANCTs was determined using the paired t-test. We considered the actual values of PCR efficiencies in the calculation of relative expression of genes. The association between patients’ data and relative expression of *SET1* and *ZFX* genes was estimated using Chi-square. The correlation between relative expressions of *SET1* and *ZFX* was appraised using the regression model. P values less than 0.05 were regarded as significant. Receiver operating characteristic (ROC) curve was depicted to assess the diagnostic power of genes. 

**Table 1:** Sequences of primers for expression assays

Gene name	Primer sequence	Primer length	Product length
*B2M*	F: AGATGAGTATGCCTGCCGTG	20	104
	R: CGGCATCTTCAAACCTCCA	19	
*SET1*	F: GAACAGGAAGAAGCGATTGAACAC	24	203
	R: GCAGACACTTGTGGATGGTTG	21	
*ZFX*	F: GGAGATGATGACTTAGGTGGAACTG	25	105
	R: GCTGGGAAGACGAATACTGCTG	22	

## Results

**Detailed information of patients**

Demographic and clinical data of patients are summarized in Table 2.

**Table 2:** Summary of demographic and clinical data of patients (for each parameter, there is a number of missing data).

Parameters		Values (%)
Gender		
	Male	21 (80.8)
	Female	5 (19.2)
Site of primary		
	Cardia	10 (37)
	Antrum	9 (33.3)
	Body	8 (29.7)
Histological grade		
	2	8 (36.4)
	3	13 (59.1)
	4	1 (4.5)
Lymphatic invasion		
	Yes	22 (81.5)
	No	5 (18.5)
Vascular invasion		
	Yes	22 (81.5)
	No	5 (18.5)
Peritoneal invasion		
	Yes	16 (59.3)
	No	11 (40.3)
TNM stage^*^		
	I	1 (3.7)
	II	8 (29.6)
	III	13 (48.2)
	IV	5 (18.5)
Smoking		
	Non-Smoker	10 (50)
	Smoker	3 (15)
	Ex-Smoker	7 (35)

* TNM: Tumor, Node, Metastasis

**Expression assays**

**Figure 1:**
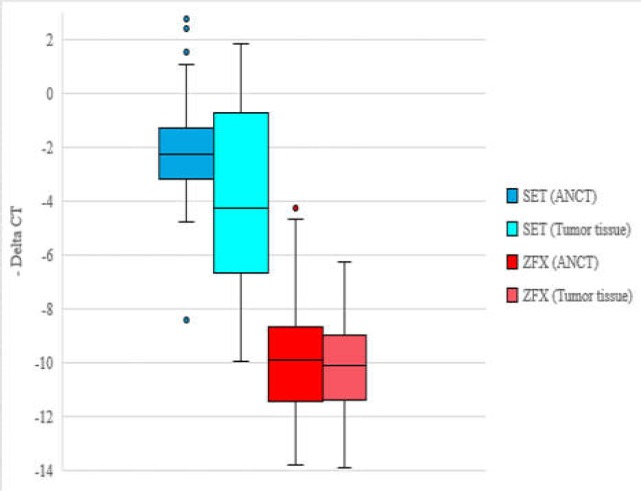
Relative expression of *SET1* and *ZFX* genes in gastric cancer samples and their pared ANCTs

*SET1* gene was down-regulated in tumoral tissues compared with ANCTs (expression ratio=0.25, P=0.015). However, the expression of *ZFX* was similar between tumoral tissues and ANCTs (expression ratio=0.97, P=0.945) (Figure 1).

Association analysis between expression levels of *SET1* and *ZFX* genes and clinical data revealed a significant association between the site of primary tumor and *SET1* relative expression in tumoral tissues versus ANCTs. Specifically, this gene was down-regulated in all tumors originating from cardia. Table 3 provides the results of association analysis.

**Table 3:** The results of association analysis between the expression of genes and clinical data (Up-/down-regulation of genes have been defined based on the relative expression of each gene in tumoral samples versus its paired ANCT)

	*SET1 *up-regulation	*SET1 *down-regulation	P value	*ZFX *up-regulation	*ZFX *down-regulation	P value
Gender			0.538			0.907
Female	2 (40%)	3 (60%)		3 (60%)	2 (40%)	
Male	5 (23.8%)	16 (76.2%)		12 (57.1%)	9 (42.9%)	
Site of primary tumor			< 0.001			0.782
Cardia	0 (0%)	10 (100%)		6 (60%)	4 (40%)	
Antrum	7 (77.8%)	2 (22.2%)		6 (66.7%)	3 (33.3%)	
Body	1 (12.5%)	7 (87.5%)		4 (50%)	4 (50%)	
Histological grade			0.521			0.621
2	1 (12.5%)	7 (87.5%)		6 (75%)	2 (25%)	
3	5 (38.5%)	8 (61.5%)		7 (53.8%)	6 (46.2%)	
4	0 (0%)	1 (100%)		1 (100%)	0 (0%)	
Lymphatic invasion			0.573			0.331
Yes	6 (27.3%)	16 (72.7%)		14 (63.6% )	8 (36.4 %)	
No	2 (40%)	3 (60%)		2 (40%)	3 (60%)	
Vascular invasion			0.573			0.331
Yes	6 (27.3%)	16 (72.7%)		14 (63.6% )	8 (36.4 %)	
No	2 (40%)	3 (60%)		2 (40%)	3 (60%)	
Peritoneal invasion			0.135			0.679
Yes	3 (18.8%)	13 (81.2%)		10 (62.5%)	6 (37.5%)	
No	5 (45.5%)	6 (54.5%)		6 (54.5%)	5 (45.5%)	
TNM^*^ Staging			0.821			0.171
I	0 (0%)	1 (100%)		0 (0%)	1 (100%)	
II	2 (25%)	6 (75%)		7 (87.5%)	1 (12.5%)	
III	5 (38.5%)	8 (61.5%)		6 (46.2%)	7 (53.8%)	
IV	1 (20%)	4 (80%)		3 (60%)	2 (40%)	
Smoking			0.988			0.321
Non-smoker	3 (30%)	7 (70%)		6 (60%)	4 (40%)	
Smoker	1 (33.3%)	2 (66.7%)		3 (100%)	0 (0%)	
Ex- smoker	2 (28.6%)	5 (71.4%)		3 (42.9%)	4 (57.1%)	

* TNM: Tumor, Node, Metastasis

**Table 4:** The results of ROC curve analysis (a: Youden index, Estimate criterion: optimal cut-off point for gene expression level)

	Estimate criterion	Area Under Curve	J^a^	Sensitivity	Specificity	P-value
*SET1*	> 4.76	0.68	0.43	46.7	96.7	0.01

**Figure 2:**
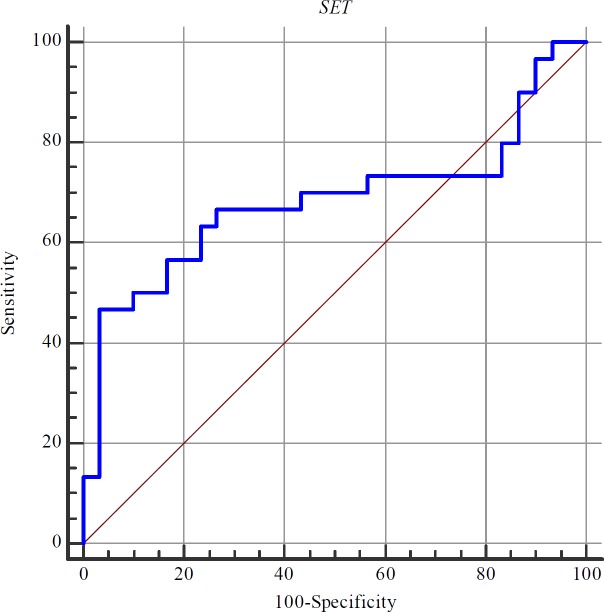
ROC curve of SET1 transcript levels for gastric cancer diagnosis (Values on X and Y axes show percentages).

**ROC curve analysis**

**Figure 3:**
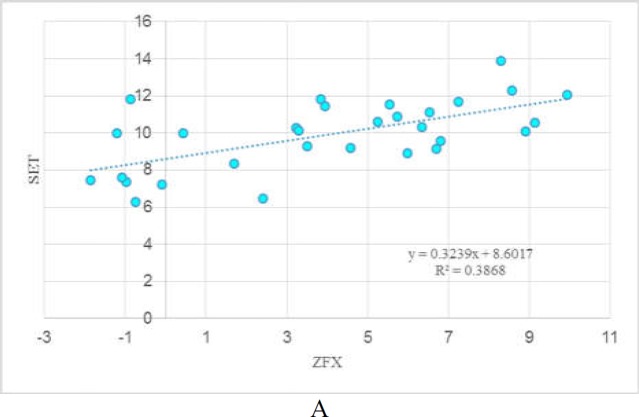
Correlation between expression levels of *SET1* and *ZFX* in tumoral tissues (A) and ANCTs (B).

Based on the area under the ROC curve, the diagnostic power of transcript levels of *SET1* in gastric cancer was 0.68 (Figure 3). Although the specificity was high (96.7%), transcript levels of *SET1* were not sensitive markers in gastric cancer (Table 4).

**Correlation between expression levels of** ***SET1*** **and** ***ZFX***

There were remarkable correlations between the expression levels of *SET1* and *ZFX* both in tumoral tissues (R^2^=0.38, P<0.05) and in ANCTs (R^2^=0.23, P<0.05). Figure 3 depicts the correlation between expression levels of *SET1* and *ZFX* in these tissues.

## Discussion

In the current project, we investigated the expression of *SET1* and *ZFX* in paired gastric tissues and reported down-regulation of *SET1* in cancerous tissues while similar expression of *ZFX* was found between two sets of tissues.

Previous studies have shown the crucial function of SET in the cell cycle progression and its involvement in the evolution of hematological malignancies, as well as breast and ovarian cancers (1, 10, 11). In early breast cancer, SET is involved in the inhibition of PP2A. Lower levels of PP2A have indicated poor outcome and doxorubicin resistance, while its over-expression implied a favorable therapeutic outcome (11). Contrary to our results, a recent study has shown the over-expression of SET in gastric tumor specimens in an animal model. Authors also reported correlations between SET expression levels and poor outcome of gastric cancer in humans. Their in vitro experiments indicated the role of SET in induction of stemness in cancer cell lines (12). The discrepancy between our results and the results of the mentioned study might be due to our small sample size or the difference in the expression analysis (real-time PCR versus Immunohistochemistry). 

We reported similar levels of *ZFX* expression in tumoral tissues and ANCTs. Our results are in accordance with the Nikpour et al. study which showed high expression of ZFX in 47% of the assessed tumor tissues and low expression of this gene in the remaining samples (8). However, a previous study has shown a tendency towards silencing of Zinc-finger proteins via promoter hypermethylation in gastric cancer (13). Another study has reported elevated expression of ZFX in colorectal tissues compared with corresponding normal tissues. Further, ZFX protein levels were associated with tumor features including differentiation level, size, invasion, metastasis, and patients’ outcome (14). A meta-analysis of eight studies has indicated over-expression of ZFX as a marker of poor prognosis particularly in colorectal cancer (15). Nevertheless, the data regarding the role of ZFX in gastric cancer are insufficient. Thus, further studies of expression of this gene at transcript and protein levels in addition to promoter methylation investigations are required to capture the function of ZFX in gastric cancer.

Association analysis between expression levels of genes and clinical data revealed no significant association except for the association between site of primary tumor and *SET1* relative expression. Notably, this gene was down-regulated in all tumors originating from cardia. A previous immunohistochemical study has shown difference in clinical features and gene expression profile between cardia and non-cardia. It supported the theory that cardia cancers belong to a definite type of gastric cancer which is distinctive from non-cardia cancers (16). Another study using Affymetrix GeneChip U133A has reported a distinct pattern of gene expression between cardia and non-cardia gastric cancers (17). Thus, our results further support the distinction between cardia and non-cardia cancers.

Although *SET1* transcript levels were considered as specific markers in gastric cancer, their sensitivity for diagnosis of cancer was low. However, based on the reported pattern of expression in cardia cancers, this gene can be a diagnostic marker in tumors originating from this region. Yet, given the low number of samples, this supposition needs to be further validated. Accordingly, the data presented in this manuscript cannot verify the biomarker potential for either of the assessed genes.

Finally, we reported significant correlations between the expression levels of *SET1* and *ZFX* in gastric tissues suggesting the role of ZFX in the regulation of SET1 expression in this tissue. However, *in vitro* studies are required to verify this supposition.

In brief, the current study provided evidence for dysregulation of *SET1* in gastric cancer tissues. It warrants future experiments to reveal the exact role and underlying mechanism of such dysregulation. Our study had the strength of simultaneous assessment of expression of a gene and its regulating transcription factor. However, the main limitation of the study was its small sample size.

## Conflict of interests

The authors declare that they have no conflict of interest.
